# Racial Disparities in Access to Maternity Care Practices That Support Breastfeeding — United States, 2011

**Published:** 2014-08-22

**Authors:** Jennifer N. Lind, Cria G. Perrine, Ruowei Li, Kelley S. Scanlon, Laurence M. Grummer-Strawn

**Affiliations:** 1 Epidemic Intelligence Service, CDC; 2 Division of Nutrition, Physical Activity, and Obesity, National Center for Chronic Disease Prevention and Health Promotion, CDC

Despite the well documented health benefits of breastfeeding (1), initiation of breastfeeding and breastfeeding duration rates among black infants in the United States are approximately 16% lower than among whites (2). Although many factors play a role in a woman’s ability to breastfeed, experiences during the childbirth hospitalization are critical for establishing breastfeeding (3). To analyze whether the implementation by maternity facilities of practices that support breastfeeding varied depending on the racial composition of the area surrounding the facility, CDC linked data from its 2011 Maternity Practices in Infant Nutrition and Care (mPINC) survey to U.S. Census data on the percentage of blacks living within the zip code area of each facility. The results of that analysis indicated that facilities in zip code areas where the percentage of black residents was >12.2% (the national average during 2007–2011) were less likely than facilities in zip code areas where the percentage was ≤12.2% to meet five of 10 mPINC indicators for recommended practices supportive of breastfeeding and more likely to implement one practice; differences for the other four practices were not statistically significant. Comparing facilities in areas with >12.2% black residents with facilities in areas with ≤12.2% black residents, the largest differences were in the percentage of facilities that implemented recommended practices related to early initiation of breastfeeding (46.0% compared with 59.9%), limited use of breastfeeding supplements (13.1% compared with 25.8%), and rooming-in (27.7% compared with 39.4%). These findings suggest there are racial disparities in access to maternity care practices known to support breastfeeding.

The mPINC survey is a biennial census of maternity facilities (hospitals and free-standing birth centers) in the United States and its territories (4). The survey is sent to the person at each facility most knowledgeable about the facility’s maternity care practices and policies. A total of 2,727 facilities participated in the 2011 mPINC survey (response rate = 83%). These data were analyzed for 10 mPINC indicators for recommended maternity care practices* from the World Health Organization/United Nations Children’s Fund’s *Ten Steps to Successful Breastfeeding* (5). The *Ten Steps* are evidence-based practices shown to increase breastfeeding exclusivity and duration, and are the basis for the Baby-Friendly Hospital Initiative.†

To estimate the prevalence of facilities with recommended maternity care practices by the percentage of black residents in their area, zip code level data for the category “non-Hispanic black or African American alone” were obtained for the period 2007–2011 from the U.S. Census Bureau’s American Community Survey (ACS). ACS is a continuous nationwide survey that collects detailed information on demographic, social, economic, and housing characteristics; these data are only available by zip code as 5-year estimates (6). ACS and mPINC data were linked by zip codes; of the 2,727 facilities that participated in the 2011 mPINC survey, 84 (3%) facilities were missing zip code level racial data in ACS, resulting in a final analytic sample of 2,643 facilities. Facilities were divided into two categories: 1) those in zip code areas where the percentage of black residents was >12.2% (the national average during 2007–2011) (6) and 2) those in zip code areas where the percentage was ≤12.2%. The z-test was used to compare data from the two categories and determine whether differences in implementation of recommended maternity care practices were statistically significant (p<0.05). No other racial or ethnic groups were examined.

In 2011, three of the 10 mPINC indicators for recommended practices were met by >75% of the 2,643 facilities surveyed. The three were providing prenatal breastfeeding education (92.7%), teaching breastfeeding techniques (90.7%), and teaching mothers how to recognize and respond to infant feeding cues (84.7%) (Table).

Facilities in zip code areas with >12.2% black residents were significantly more likely to assess staff competency than facilities in zip code areas with ≤12.2% black residents (59.4% compared with 53.2%) (Table). However, facilities in zip code areas with >12.2% black residents were significantly less likely than facilities in zip code areas with ≤12.2% black residents to meet five of the nine other mPINC indicators for recommended practices: early initiation of breastfeeding (46.0% compared with 59.9%), limited use of breastfeeding supplements (13.1% compared with 25.8%), rooming-in (27.7% compared with 39.4%), limited use of pacifiers, (30.5% compared with 37.9%), and post-discharge support (23.9% compared with 29.9%) (Table).

## Discussion

In 2011, implementation of 10 recommended maternity care practices supportive of breastfeeding among 2,643 maternity facilities varied widely, ranging from 18.9% to 92.7%, and was <50% for five practices. For half of the 10 practices, implementation was significantly lower among facilities in zip code areas with a higher percentage of black residents. These findings are important because research has shown that U.S. residents usually are admitted to hospitals within a relatively short distance of where they live, although persons living in rural areas might travel farther than those in cities (7). Therefore, women living in zip code areas with a higher percentage of blacks might have less access to facilities implementing recommended maternity care practices, which might contribute to lower breastfeeding rates among blacks compared with other racial groups.

What is already known on this topic?

Breastfeeding has many health benefits for infants, yet there are persistent gaps in breastfeeding rates between black and white infants in the United States. Maternity care practices experienced during the hospital stay have a major impact on the establishment of breastfeeding.

What is added by this report?

Facilities located in zip code areas with higher percentages of blacks were less likely to meet five indicators for recommended maternity care practices supportive of breastfeeding and more likely to meet one indicator, than facilities in areas with a lower percentage of blacks. The largest differences were for indicators related to early initiation of breastfeeding, limited use of breastfeeding supplements, and rooming-in.

What are the implications for public health practice?

Interventions are needed to ensure that all maternity care facilities are implementing the recommended policies and practices known to be important for the establishment of breastfeeding. Facilities located in areas with higher percentages of blacks might need additional support.

The reasons for the differences in maternity care practices by racial composition of the areas are not clear. Further research is needed on barriers to implementing recommended practices in these areas, on whether poorer maternity care practices are linked to lower breastfeeding rates in these areas, and on evaluating other factors that might be contributing to these disparities.

This is the first report based on national data showing that practices at maternity facilities vary with the racial composition of the zip code area in which the facility is located. However, similar findings were observed in a previous study in North Carolina that assessed whether there were differences in breastfeeding support services available through the Supplemental Nutrition Program for Women, Infants, and Children (WIC) program based on the county level racial/ethnic composition of the WIC sites. It was found not only that breastfeeding initiation by WIC site was negatively associated with the percentage of black clients, but also that WIC sites with higher percentages of black clients were less likely to offer clinic-based breastfeeding support services (8).

In a review of U.S.-based randomized trials evaluating breastfeeding interventions targeting minorities, interventions to change hospital or WIC policies, including enhanced practices and services, were among the public health approaches found to successfully improve breastfeeding outcomes among minority women (9). CDC currently is funding a project that addresses the need for quality improvement in maternity care practices. In June 2012, CDC awarded a 3-year cooperative agreement to the National Initiative for Children’s Healthcare Quality to assist 89 hospitals, mostly located in states that have lower breastfeeding rates and that serve low-income and minority women, with improving maternity care practices to support breastfeeding and to move toward the Baby-Friendly designation. Detailed descriptions of the cooperative agreement program have been published (2,10).

The findings in this report are subject to at least four limitations. First, one mPINC indicator for each of the *Ten Steps* was selected; these indicators are consistent with the *Ten Steps*,§ but might not encompass all aspects of each step. Second, although the mPINC survey was sent to the person identified as the most knowledgeable about the facility’s policies and practices and facilities were encouraged to get input from key staff members as needed, responses might not accurately reflect actual practices. Third, the racial composition of the patients served at each facility is not collected in the mPINC survey. However, because most U.S. residents are admitted to hospitals close to where they live and most hospital service areas have only one local hospital, the data in this report for zip code areas are likely reasonable estimates for the racial composition of hospital patients, assuming overall hospital admission patterns (7) apply to births. Finally, only facilities with zip code level race data were included in this analysis. Excluded facilities might have had different percentages of blacks and maternity care practices. However, only 3% of facilities were excluded, which is not likely to have affected results.

The findings suggest that the implementation of maternity care practices supportive of breastfeeding vary based on the racial composition of the area, which means women living in areas with higher percentages of blacks might have less access to these services. Although the reasons for these disparities are unclear, the results might provide some insight into why there has been a persistent gap in breastfeeding initiation and duration rates between black and white infants in the United States. All facilities, regardless of the racial/ethnic composition of the populations they serve, can support the breastfeeding decisions of their patients by implementing evidence-based policies and practices shown to be critical for establishing breastfeeding, so that more infants are able to reap the numerous health benefits of breastfeeding.

## Figures and Tables

**TABLE:** Prevalence of facilities meeting indicators for recommended maternity care practices,* by racial composition† of the zip code areas where the facilities were located — Maternity Practices in Infant Nutrition and Care Survey (mPINC), United States, 2011

mPINC indicators for recommended maternity care practices	Total facilities surveyed (N = 2,643§)	Percentage of black residents in the facility zip code area				

		≤12.2% (n = 2,030§)	>12.2% (n = 613§)	Percentage-point difference	Standard error of the difference	p-value
		
	%	%	%			
Model breastfeeding policy: hospital has a written breastfeeding policy that includes 10 model policy elements.	18.9	18.5	20.3	−1.8	1.87	0.33
Staff competency assessment: nurses/birth attendants are assessed for competency in basic breastfeeding management and support at least once per year.	54.6	53.2	59.4	−6.2	2.28	0.01¶
Prenatal breastfeeding education: breastfeeding education is included as a routine element of prenatal classes.	92.7	92.9	91.8	1.1	1.25	0.38
Early initiation of breastfeeding: ≥90% of healthy, full-term, breastfed infants initiate breastfeeding within 1 hour of uncomplicated vaginal birth.	56.7	59.9	46.0	13.9	2.31	<0.01¶
Teach breastfeeding techniques: ≥90% of mothers who are breastfeeding or intend to breastfeed are taught breastfeeding techniques (e.g., positioning and how to express milk).	90.7	91.2	89.2	2.0	1.41	0.16
Limited use of breastfeeding supplements: <10% of healthy, full-term, breastfed infants are supplemented with formula, glucose water, or water.	22.8	25.8	13.1	12.7	1.69	<0.01¶
Rooming-in: ≥90% of healthy, full-term infants, regardless of feeding method, remain with their mother for at least 23 hours per day during the hospital stay.	36.7	39.4	27.7	11.7	2.12	<0.01¶
Teach feeding cues: ≥90% of mothers are taught to recognize and respond to infant feeding cues instead of feeding on a set schedule.	84.7	85.1	83.2	1.9	1.71	0.26
Limited use of pacifiers: <10% of healthy, full-term, breastfed infants are given pacifiers by maternity care staff members.	36.2	37.9	30.5	7.4	2.16	<0.01¶
Post-discharge support: hospital routinely provides three modes of post-discharge support to breastfeeding mothers (physical contact, active reaching out, and referrals).	28.5	29.9	23.9	6.0	2.00	<0.01¶

* mPINC indicators for recommended maternity care practices are from *Ten Steps to Successful Breastfeeding*, available at http://www.babyfriendlyusa.org/about-us/babyfriendly-hospital-initiative/the-ten-steps.

† Zip code areas in which the percentage of “non-Hispanic black or African American” residents was >12.2% (the national average during 2007–2011), compared with ≤12.2%, according to data from the U.S. Census Bureau’s American Community Survey.

§ Number of respondents varied slightly from the total for each of the prevalence estimates.

¶ Statistically significant percentage-point difference by z-test.

## Footnotes

* The 10 mPINC indicators for recommended maternity care practices from the *Ten Steps* were as follows: 1) Model breastfeeding policy: hospital has a written breastfeeding policy that includes 10 model policy elements; 2) Staff competency assessment: nurses/birth attendants are assessed for competency in basic breastfeeding management and support at least once per year; 3) Prenatal breastfeeding education: breastfeeding education is included as a routine element of prenatal classes; 4) Early initiation of breastfeeding: ≥90% of healthy, full-term, breastfed infants initiate breastfeeding within 1 hour of uncomplicated vaginal birth; 5) Teach breastfeeding techniques: ≥90% of mothers who are breastfeeding or intend to breastfeed are taught breastfeeding techniques (e.g., positioning and how to express milk); 6) Limited use of breastfeeding supplements: <10% of healthy, full-term, breastfed infants are supplemented with formula, glucose water, or water; 7) Rooming-in: ≥90% of healthy, full-term infants, regardless of feeding method, remain with their mother for at least 23 hours per day during the hospital stay; 8) Teach feeding cues: ≥90% of mothers are taught to recognize and respond to infant feeding cues instead of feeding on a set schedule; 9) Limited use of pacifiers: <10% of healthy, full-term, breastfed infants are given pacifiers by maternity care staff members; and 10) Post-discharge support: hospital routinely provides three modes of post-discharge support to breastfeeding mothers (physical contact, active reaching out, and referrals).

† Additional information available at http://www.babyfriendlyusa.org/about-us/baby-friendly-hospital-initiative/the-ten-steps.

§ Additional information available at https://www.babyfriendlyusa.org/get-started/the-guidelines-evaluation-criteria.
